# Shorter TB treatment regimens should be safer as well

**DOI:** 10.5588/pha.23.0026

**Published:** 2023-09-21

**Authors:** O. Rucsineanu, P. Agbassi, R. Herrera, M. Low, L. McKenna, J. Stillo, P. Winarni, A. Acharya, A. H. Sari

**Affiliations:** 1 Moldova National Association of Tuberculosis Patients “SMIT” (Society of Moldova against Tuberculosis), Balti, Moldova; 2 Global TB Community Advisory Board, New York, NY, USA; 3 Department of Computer Science, University of Cape Town, Cape Town, South Africa; 4 Treatment Action Group, New York, NY, USA; 5 Department of Anthropology, Wayne State University, Detroit, MI, USA; 6 Independent Consultant, Mumbai, India

**Keywords:** safer, shorter, toxicity, person-centred

## Abstract

Most ongoing and planned TB therapeutic trials are focused on shortening the duration of treatment while giving less consideration to other aspects of TB care that are important to people with TB. Here we argue that other variables besides duration of TB treatment should also be considered when developing new TB treatment regimens, including drug toxicity, time spent in monitoring and overall quality of life while on therapy. We examine the specific use of linezolid in treatment-shortening trials for drug-susceptible TB and propose additional endpoints that should be prioritised in TB treatment studies.

## Abstract (fr)

La majorité des essais thérapeutiques en cours et prévus sur la TB se concentrent sur la réduction de la durée du traitement tout en accordant moins d’attention à d’autres aspects des soins de la TB qui sont importants pour les personnes atteintes de la TB. Nous soutenons ici que d’autres variables que la durée du traitement de la TB devrait également être prises en compte lors de l’élaboration de nouveaux schémas thérapeutiques, notamment la toxicité des médicaments, le temps passé à la surveillance et la qualité de vie globale pendant le traitement. Nous examinons l’utilisation spécifique du linézolide dans les essais de raccourcissement du traitement de la TB sensible aux médicaments et proposons des critères d'évaluation supplémentaires qui devraient être prioritaires dans les études sur le traitement de la TB.

Each year more than 10 million people fall ill with TB and more than a million die from the disease.^1^ The past decade has witnessed remarkable transformations in the way that TB is prevented and treated.^2^ Thanks to the dedicated efforts of TB researchers and clinicians working in partnership with impacted communities, it is now possible to prevent TB in as little as 1 month, treat drug-susceptible forms of TB disease (DS-TB) in 4 months and treat drug-resistant forms of TB (DR-TB) in 6 months. These accomplishments are notable after years of stagnation in TB trials and are due, in part, to concerted advocacy by the global TB community.^3^

Even with the advances in TB care, treatment regimens are fraught with challenges and considered burdensome to people with the disease. Ongoing and planned TB treatment research initiatives are focused on further optimising therapeutic duration or route of administration, with ‘treatment shortening’ of all-oral regimens driving the agenda for a majority of ongoing and proposed clinical studies, especially those on DS-TB.^4^ While there is evidence suggesting that treatment duration is a factor associated with the ability of a person to successfully complete treatment for a variety of medical conditions, including TB, there are other important issues to consider. These include, but are not limited to, therapeutic trust, pill burden, drug–drug interactions, treatment literacy and other competing socio-economic and medical needs.^5^ One key factor in determining whether an individual can take a drug as prescribed is the experience of the ‘side effects’ or adverse events associated with a drug. Multiple studies show that adverse events drive therapeutic challenges for many health conditions, including TB.^6^ Studies conducted among people with TB show that as many as half of those who do not complete treatment cite the experience of adverse events as the main reason for discontinuation.^7^ Because the experience of adverse events may be equally or more important than treatment duration in determining who finishes a course of prescribed TB therapy, optimising regimens for safety and tolerability should be a priority, alongside efficacy and duration in ongoing and planned initiatives and studies for the evaluation of the next generation of TB treatment regimens. This is not a novel concept in TB trials (e.g., ZeNix; NCT03086486) and is one reason why some drugs that are good at killing TB bacteria but have challenging side effects (e.g., pyrazinamide [PZA]) are given for shorter periods of time than the rest of the treatment regimen.^8^

All drugs and treatment regimens are associated with adverse effects, including those currently in use for DS-TB. The 6-month regimen of isoniazid, rifampicin, PZA and ethambutol can cause hepatotoxicity, optic neuropathy and peripheral neuropathy, although less frequently than linezolid (LZD) and which can be prevented with pyridoxine. The recently WHO-recommended 4-month regimen of isoniazid, rifapentine, moxifloxacin and PZA, which has been shown to be non-inferior to the standard 6-month regimen, both in terms of efficacy and safety, can also cause prolongation of the QTcF interval in additional to hepatotoxicity and peripheral neuropathy that is preventable with pyridoxine.^9^ Most novel regimens for DS-TB will be compared to one of these two standard regimens and will need to demonstrate equivalent efficacy, tolerability and safety, and while researchers might be willing to accommodate similar side effects in exchange for shorter treatment durations, the same might not be true of members and representatives of impacted communities. Affected communities may perceive and value trade-offs differently from researchers, clinicians and donors. It is therefore essential to consult impacted communities on these matters.

By optimising regimens with a myopic focus on treatment duration, researchers and clinicians are missing opportunities to address other aspects of treatment that are challenging for people with TB. It is also worth noting that the concept of treatment duration appears to have been narrowly construed by clinical researchers, focusing only on the amount of time a treatment regimen must be administered. It overlooks, for example, the amount of time people on treatment need to spend engaging in all aspects of care, including time spent feeling unwell, more frequent visits to healthcare providers for monitoring, out-of-pocket expenses and time spent waiting for test results. Therefore, while a treatment regimen may have a shorter overall duration, it is crucial to consider the additional time individuals may have to spend at the clinic for examinations, procedures or adjustments to the regimen. This could potentially make the regimen more burdensome in terms of the time dedicated to treatment-related activities. It is essential to include such measures as co-primary endpoints in TB trials, as they hold significant importance in the lives of individuals undergoing therapy. These measures could be modelled after the concept of ‘home time,’ based on the number of days spent at home without in-person healthcare interactions. The concept of ‘home time’ has already been utilised as an outcome measure in studies related to cancer, heart disease and stroke.^10^

LZD is an oxazolidinone antibiotic that has been shown to be effective against *Mycobacterium tuberculosis*, enabling treatment shortening for DR-TB.^11^ Unfortunately, in addition to its efficacy, LZD is associated with notable toxicity, including bone marrow suppression, peripheral neuropathy, optic neuritis and lactic acidosis.^12^ Even when used in treating DR-TB, LZD has to be discontinued for toxicity reasons in as many as one in every five (20%) persons receiving it^13^ (see Table 1 for one DR-TB survivor’s experience taking LZD). Nevertheless, because of its ability to kill TB bacteria, LZD and other new drugs from the same class are of great interest to researchers seeking to further shorten treatment for both DR- and DS-TB. High rates of toxicity seen with LZD in DR-TB studies are a cause for concern among impacted communities, especially given its recent and planned inclusion in treatment-shortening trials of DS-TB regimens, for which the risk tolerance and acceptability of adverse events is much lower.

**TABLE 1:** The experience of taking linezolid for drug-resistant TB

In May 2021, I (Akshata Acharya) woke up one day realising that I have a small swelling below my right ear. The diagnosis took almost a month and finally it was revealed that I have multidrug-resistant TB, with resistance to rifampicin… The doctors put me on a 9-month shorter regimen. The high dose did not suit my body and I kept getting worse day by day. I was throwing up 4 times a day, my appetite was lost, my body was covered in boils and rashes, I had severe burning sensation in my stomach, my joints were wrecked ultimately, and I was dependent on my parents… My parents took me to the hospital and after seeing me in a horrible condition, the doctors changed my course from 9-month shorter regimen to 18–24 months all oral longer regimen. I was hesitant to increase the duration of such ‘torturous treatment’ and hence kept resisting. But the doctors made me look at the brighter side of it, that is to have lesser number of tablets in a day. From 22 tablets a day, I was going to come down to having just 8–9 tablets each day. I did show some improvement but once again after 3 months, I started showing a declining trend of haemoglobin, but the drug was not stopped right away. Six months after taking linezolid, I developed severe peripheral neuropathy. I observed a weird tingling sensation in my feet, which drastically increased with each passing day. I was finally put in a wheelchair as I lost my mobility. All of this happened over a span of one week- the doctors confirmed that I had developed severe peripheral neuropathy which was induced by linezolid. I was hospitalised, yet again. All my drugs were put on hold. I used to scream all day, even more during the nights because of the excruciating pain. I was sent home after a week as the doctors said reversing neuropathy would be extremely lengthy process. I resumed my TB medicines and luckily, I have been able to tolerate them ever since. I eventually got off the wheelchair and slowly started practicing my walk. As of today, I am still struggling with neuropathy. It’s been a year I haven’t worn shoes or socks. I cannot jump or run either. My TB treatment has been exceptionally hard considering the complications that came up due to peripheral neuropathy.

One recently completed study, the TRUNCATE TB trial, assessed LZD in 8-week regimens for DS-TB (NCT03474198).^14^ Results showed that an 8-week regimen consisting of bedaquiline, LZD (administered at 600 mg daily), isoniazid, PZA and ethambutol had similar rates of unsatisfactory outcomes and similar rates of serious and severe adverse events when compared with the standard 6-month regimen for treating DS-TB. The safety assessment, however, did not report on the occurrence of mild or moderate adverse events that may be troubling to participants, including peripheral neuropathy and optic neuritis. These events can be problematic for the people experiencing them, even if they do not meet trial-specified criteria for serious or severe events used by trial investigators. Current toxicity assessments in most trials of TB treatment shortening also overlook the subjective experience of adverse events—more attention must be given to the amount of time people spend feeling unwell, the required toxicity monitoring schedules and the relative impact adverse events of any grade may have on the quality of life of the person on therapy. These factors are not adequately captured in current adverse event grading systems.^15^ It is also worth noting that persons at risk for the development of LZD-associated adverse events were excluded from the study and will likely be excluded from future studies of shorter regimens that include this drug, meaning the risks associated with LZD use in general practice will be under-estimated compared to real world occurrence.

Shorter regimens present many potential desirable benefits to people with TB and their communities, including less time out of work or school, more ability to resume family care and other responsibilities, etc. People with TB want more than just shorter regimens and have real concerns about toxicity. While our focus here is primarily on LZD, it is crucial to subject other drugs undergoing evaluation in TB clinical trials to similar scrutiny. Specifically, clofazimine, which is used for DS-TB, should be examined due to the potential risk of skin hyperpigmentation leading to inadvertent disclosure and stigma. Additionally, injectable agents used for DR-TB should also be carefully assessed due to the associated risk of permanent hearing loss. We call for the inclusion of more person-centred endpoints in TB treatment trials, including those that measure time toxicity such as ‘home days’ and compound efficacy/safety outcomes such as ‘cured without any significant adverse events’, and follow-up for long-term resolution of side effects that last beyond the end of treatment. We also call for a deeper examination for reasons why people are unable to complete treatment, including whether or not their experience of side effects drove their decision to discontinue treatment. ‘Shorter’ in terms of overall therapeutic duration is not always better, especially when the drugs used to shorten treatment may be associated with increased risk of toxicity or require more intensive monitoring to assess for possible toxicity. More work is needed to better define the qualitative experience of being treated for TB and to focus on aspects of the treatment journey that matter most to people with the disease (see Table 2). Treatment-shortening studies and shorter regimens that fail to address other meaningful aspects of care identified by people with TB will fall short of what is needed to end TB.

**TABLE 2:** Additional considerations in treatment shortening trials

Time spent engaging in the activities of treatment (such as monitoring, regimen adjustment, time spent feeling unwell); Time out of work or school, or unable to meet household or other responsibilities and their economic implications; Type of adverse events and qualitative experience of them (such as pain, reversibility, impact on other social roles); Predictability of adverse event, including predisposing risk factors; Preferences of person being treated; Factors driving loss to follow-up

## References

[1] World Health Organization (2022). Global TB Report, 2022.

[2] McKenna L (2023). The 1/4/6x24 campaign to cure tuberculosis quickly. Nat Med.

[3] LaHood A (2022). Comparing timelines and evidence available to support new TB, HIV, and HCV drug approvals: the same, only different. PLoS One.

[4] Spellberg B (2016). The new antibiotic mantra: “shorter is better”. JAMA Intern Med.

[5] Munro SA (2007). Patient adherence to tuberculosis treatment: a systematic review of qualitative research. PLoS Med.

[6] Awofeso N (2008). Anti-tuberculosis medication side-effects constitute major factor for poor adherence to tuberculosis treatment. Bull World Health Organ.

[7] Rucs¸ineanu O, Stillo J, Ates¸ V (2018). Assessing the satisfaction level of tuberculosis patients in regards to medical services and community support during treatment.

[8] Zhang N (2022). Optimizing pyrazinamide for the treatment of tuberculosis. Eur Respir J.

[9] Dorman S (2021). Four-month rifapentine regimens with or without moxifloxacin for tuberculosis. N Engl J Med.

[10] Banerjee R, George M, Gupta A (2023). Maximizing home time for persons with cancer. JCO Oncol Pract.

[11] World Health Organization (2020). WHO consolidated guidelines on tuberculosis: module 4: treatment: drug-resistant tuberculosis treatment.

[12] Zhang X (2015). Systematic review and meta-analysis of the efficacy and safety of therapy with linezolid containing regimens in the treatment of multidrug-resistant and extensively drug-resistant tuberculosis. J Thorac Dis.

[13] Wasserman S (2022). Linezolid toxicity in patient with drug-resistant tuberculosis: a prospective cohort study. J Antimicrob Chemother.

[14] Paton N (2023). Treatment strategy for rifampicin-susceptible tuberculosis. N Engl J Med.

[15] Thanarajasingam G (2016). Longitudinal adverse event assessment in oncology clinical trials: the toxicity over time (ToxT) analysis of Alliance trials NCCTG N9741 and 979254. Lancet Oncol.

